# Supplementary material for: Dynalign II: common secondary structure prediction for RNA homologs with domain insertions
Source: Nucleic Acids Res. 2014 Nov 21;42(22):13939–48. doi: 10.1093/nar/gku1172 (PMC4267632; doi:10.1093/nar/gku1172)
Supplement: SUPPLEMENTARY DATA [file supp_gku1172_nar-02021-z-2014-File012.zip › manual/javadoc/RNAstructure_java_utilities/FileChooser.html]

FileChooser


JavaScript is disabled on your browser.


- Overview
- Package
- Class
- Tree
- Index
- Help

- Prev Class
- Next Class

- Frames
- No Frames

- All Classes

- Summary:
- Nested |
- Field |
- Constr |
- Method

- Detail:
- Field |
- Constr |
- Method


RNAstructure\_java\_utilities

## Class FileChooser

- java.lang.Object
- - RNAstructure\_java\_utilities.FileChooser

- All Implemented Interfaces:
  :   Serializable

  ---

    

  ```
  public class FileChooser
  extends Object
  implements Serializable
  ```

  A class that creates a file chooser so the user can select a file to open
  or save.

  See Also:
  :   Serialized Form

- - ### Nested Class Summary

    Nested Classes

    | Modifier and Type | Class and Description |
    | --- | --- |
    | `private static class` | `FileChooser.Types` An enum that holds the possible values for the file chooser type. |
  - ### Field Summary

    Fields

    | Modifier and Type | Field and Description |
    | --- | --- |
    | `private String` | `defaultFile` The default file name for this file chooser, if necessary. |
    | `private FileChooser.Types` | `selectorType` The type of file type selector. |
    | `private static long` | `serialVersionUID` |
  - ### Constructor Summary

    Constructors

    | Modifier | Constructor and Description |
    | --- | --- |
    | `private` | `FileChooser(FileChooser.Types type, String file)` Constructor. |
  - ### Method Summary

    Methods

    | Modifier and Type | Method and Description |
    | --- | --- |
    | `static FileChooser` | `doOpen()` Create a file chooser that selects a file to open. |
    | `static FileChooser` | `doSave()` Create a file chooser that selects a file to save. |
    | `static FileChooser` | `doSave(String file)` Create a file chooser that selects a file to save, and will have a default file filled in. |
    | `String` | `getAlignment()` Select an alignment file. |
    | `String` | `getBracket()` Select a dot bracket file. |
    | `String` | `getConstraints()` Select a constraints file. |
    | `String` | `getCT()` Select a CT file. |
    | `String` | `getDotPlot()` Select a dot plot file. |
    | `String` | `getDynalign()` Select a Dynalign save file. |
    | `String` | `getFolding()` Select a folding save file. |
    | `String` | `getHelix()` Select a structure helix file. |
    | `String` | `getList()` Select an oligo list file. |
    | `String` | `getOUT()` Select a general OUT file. |
    | `String` | `getPartition()` Select a partition function save file. |
    | `String` | `getPostscript()` Select a Postscript image file. |
    | `String` | `getReport()` Select an oligo report file. |
    | `String` | `getSequence()` Select a sequence file. |
    | `String` | `getSequenceExtended()` Select a sequence file in its extended context, with more than its usual possible file types. |
    | `String` | `getSHAPE()` Select a SHAPE file. |
    | `String` | `getSVG()` Select an SVG file. |
    | `private String` | `select(String[] fileTypes, String[] extensions)` Select a file that has multiple possible input or output types. |
    | `private String` | `select(String fileType, String extension)` Select a file that has one possible input or output type. |

    - ### Methods inherited from class java.lang.Object

      `clone, equals, finalize, getClass, hashCode, notify, notifyAll, toString, wait, wait, wait`

- - ### Field Detail


    - #### defaultFile

      ```
      private String defaultFile
      ```

      The default file name for this file chooser, if necessary.


    - #### selectorType

      ```
      private FileChooser.Types selectorType
      ```

      The type of file type selector.


    - #### serialVersionUID

      ```
      private static final long serialVersionUID
      ```

      See Also:
      :   Constant Field Values
  - ### Constructor Detail


    - #### FileChooser

      ```
      private FileChooser(FileChooser.Types type,
                 String file)
                   throws IllegalArgumentException
      ```

      Constructor.

      Parameters:
      :   `type` - The type of file selector.
      :   `file` - The default file in this chooser.

      Throws:
      :   `IllegalArgumentException`
  - ### Method Detail


    - #### doOpen

      ```
      public static FileChooser doOpen()
      ```

      Create a file chooser that selects a file to open.

      Returns:
      :   The file chooser.


    - #### doSave

      ```
      public static FileChooser doSave()
      ```

      Create a file chooser that selects a file to save.

      Returns:
      :   The file chooser.


    - #### doSave

      ```
      public static FileChooser doSave(String file)
      ```

      Create a file chooser that selects a file to save, and will have a
      default file filled in.

      Parameters:
      :   `file` - The default file name.

      Returns:
      :   The file chooser.


    - #### getAlignment

      ```
      public String getAlignment()
      ```

      Select an alignment file.

      Returns:
      :   The selected alignment file.


    - #### getBracket

      ```
      public String getBracket()
      ```

      Select a dot bracket file.

      Returns:
      :   The selected dot bracket file.


    - #### getConstraints

      ```
      public String getConstraints()
      ```

      Select a constraints file.

      Returns:
      :   The selected constraints file.


    - #### getCT

      ```
      public String getCT()
      ```

      Select a CT file.

      Returns:
      :   The selected CT file.


    - #### getDotPlot

      ```
      public String getDotPlot()
      ```

      Select a dot plot file.

      Returns:
      :   The selected dot plot file.


    - #### getDynalign

      ```
      public String getDynalign()
      ```

      Select a Dynalign save file.

      Returns:
      :   The selected Dynalign save file.


    - #### getFolding

      ```
      public String getFolding()
      ```

      Select a folding save file.

      Returns:
      :   The selected folding save file.


    - #### getHelix

      ```
      public String getHelix()
      ```

      Select a structure helix file.

      Returns:
      :   The selected structure helix file.


    - #### getList

      ```
      public String getList()
      ```

      Select an oligo list file.

      Returns:
      :   The selected oligo list file.


    - #### getOUT

      ```
      public String getOUT()
      ```

      Select a general OUT file.

      Returns:
      :   The general OUT file.


    - #### getPartition

      ```
      public String getPartition()
      ```

      Select a partition function save file.

      Returns:
      :   The partition function save file.


    - #### getPostscript

      ```
      public String getPostscript()
      ```

      Select a Postscript image file.

      Returns:
      :   The Postscript image file.


    - #### getReport

      ```
      public String getReport()
      ```

      Select an oligo report file.

      Returns:
      :   The oligo report file.


    - #### getSequence

      ```
      public String getSequence()
      ```

      Select a sequence file.

      Returns:
      :   The sequence file.


    - #### getSequenceExtended

      ```
      public String getSequenceExtended()
      ```

      Select a sequence file in its extended context, with more than its
      usual possible file types.

      Returns:
      :   The sequence file.


    - #### getSHAPE

      ```
      public String getSHAPE()
      ```

      Select a SHAPE file.

      Returns:
      :   The SHAPE file.


    - #### getSVG

      ```
      public String getSVG()
      ```

      Select an SVG file.

      Returns:
      :   The SVG file.


    - #### select

      ```
      private String select(String[] fileTypes,
                  String[] extensions)
      ```

      Select a file that has multiple possible input or output types.

      Parameters:
      :   `fileTypes` - The types of file to select.
      :   `extensions` - The extensions of the file types.

      Returns:
      :   The name of the selected file.


    - #### select

      ```
      private String select(String fileType,
                  String extension)
      ```

      Select a file that has one possible input or output type.

      Parameters:
      :   `fileType` - The type of file to select.
      :   `extension` - The extension of the file types

      Returns:
      :   The name of the selected file.


- Overview
- Package
- Class
- Tree
- Index
- Help

- Prev Class
- Next Class

- Frames
- No Frames

- All Classes

- Summary:
- Nested |
- Field |
- Constr |
- Method

- Detail:
- Field |
- Constr |
- Method
